# P-1113. Comparison of Tuberculosis Exposure Frequency and Latent Tuberculosis Infection Rates Among Healthcare Workers by Occupational Classification

**DOI:** 10.1093/ofid/ofaf695.1308

**Published:** 2026-01-11

**Authors:** Seran Cheon, Si-Ho Kim, Cheon Hoo Jeon, Yu Mi Wi

**Affiliations:** Samsung Changwon Hospital, Changwon, Kyongsang-namdo, Republic of Korea; Division of Infectious Diseases, Samsung Changwon Hospital, Sungkyunkwan University, Changwon, Kyongsang-namdo, Republic of Korea; Samsung Changwon Hospital, Changwon, Kyongsang-namdo, Republic of Korea; Samsung Changwon Hospital, Changwon, Kyongsang-namdo, Republic of Korea

## Abstract

**Background:**

The Korea Disease Control and Prevention Agency classifies healthcare workers (HCWs) into five groups based on *Mycobacterium tuberculosis* exposure risk and transmission impact, guiding initial and periodic TB screening. These classifications serve as the basis for initial and periodic TB screening. However, data regarding the actual exposure frequency and the incidence of latent tuberculosis infection (LTBI) in each classification group remain limited. This study aimed to analyze the frequency of TB exposure and the incidence of LTBI among HCWs according to their occupational classification.

**Methods:**

This retrospective cohort study included HCWs at a tertiary hospital (2023–2024). Baseline LTBI screening was conducted for all staff, with annual testing only for Groups 1–4. TB exposure history and frequency were recorded. LTBI incidence was assessed in HCWs with a negative test in 2023 who underwent repeat testing in 2024.

**Results:**

Among 2,116 HCWs (27.7% male, mean age 36), TB exposure rates increased with risk classification (Group 1: 31.9%, Group 2: 17.9%, Group 3: 11.2%, Group 4: 0.6%, Group 5: 0%; P< 0.001), as did mean exposure events per person (Group 1: 0.67, Group 2: 0.37, Group 3: 0.16, Group 4: 0.01, Group 5: 0; P< 0.001). LTBI incidence did not significantly differ by classification (Group 1: 0.8%, Group 2: 0.3%, Group 3: 1.2%, Group 4: 1.0%; P< 0.001) but showed an increasing trend with exposure frequency (No exposure: 0.7%, One exposure: 0.6%, Two exposures: 2.2%, Three or more: 3.8%; P=0.065).

**Conclusion:**

This study suggests that occupational classification effectively predicts the frequency of TB exposure among HCWs. However, LTBI incidence appears to be more closely associated with the actual number of exposure events rather than occupational classification alone.

**Disclosures:**

All Authors: No reported disclosures

